# Supplementary material for: Accurate Prognosis Prediction of Pancreatic Ductal Adenocarcinoma Using Integrated Clinico-Genomic Data of Endoscopic Ultrasound-Guided Fine Needle Biopsy
Source: Cancers (Basel). 2021 Jun 3;13(11):2791. doi: 10.3390/cancers13112791 (PMC8199936; doi:10.3390/cancers13112791)
Supplement: Supplementary file 1 [file cancers-13-02791-s001.zip › cancers-1199860-supplementary/supplementary figures.docx]

**Supplementary Materials:**

Accurate Prognosis Prediction of Pancreatic Ductal Adenocarcinoma using Integrated Clinico-Genomic Data of Endoscopic Ultrasound-Guided Fine Needle Biopsy

**Table S1.** DNA QC.

**Table S2.** Sequencing QC stat.

**Table S3.** CancerSCAN gene list.


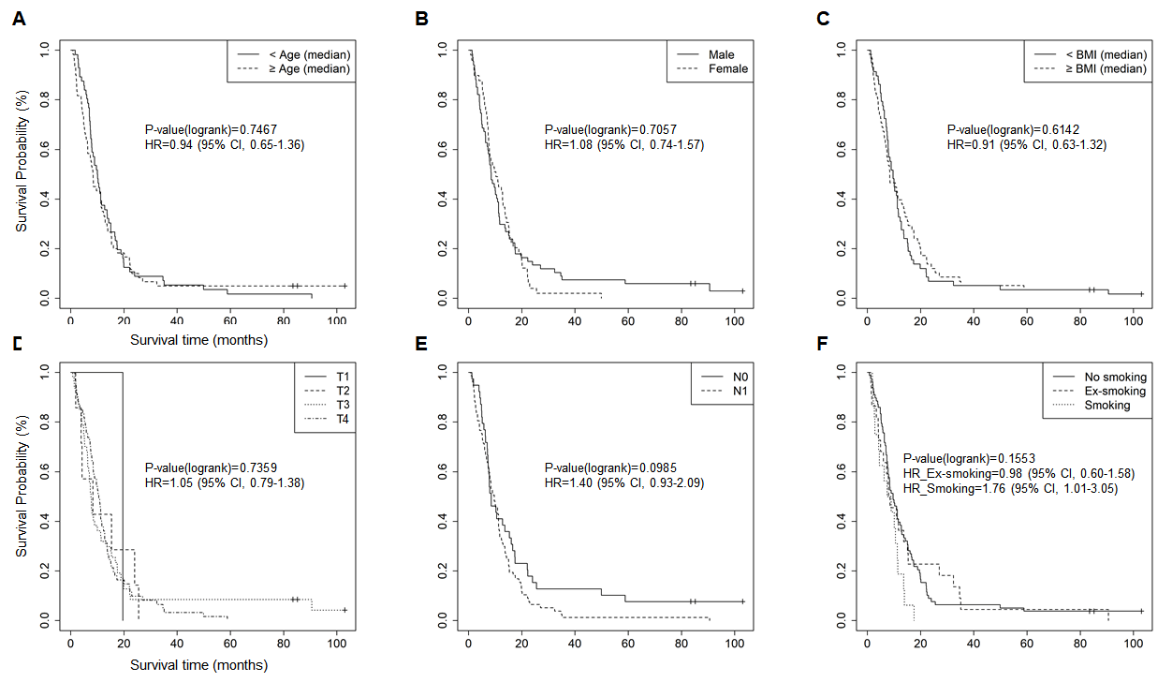


**Figure S1. Kaplan-Meier plots for clinical factors not significantly associated with survival.** The Kaplan-Meier plots demonstrate clinical factors without a significant (p<0.05) association with the survival of patients with PDAC. (A) Age, (B) Sex, (C) BMI, (D) T classification (size and direct extent of the primary tumor), (E) N classification and (F) Chemotherapy. Hazard ratio (HR), confidence interval (CI), and P value were obtained from Univariate Cox proportional hazard test.


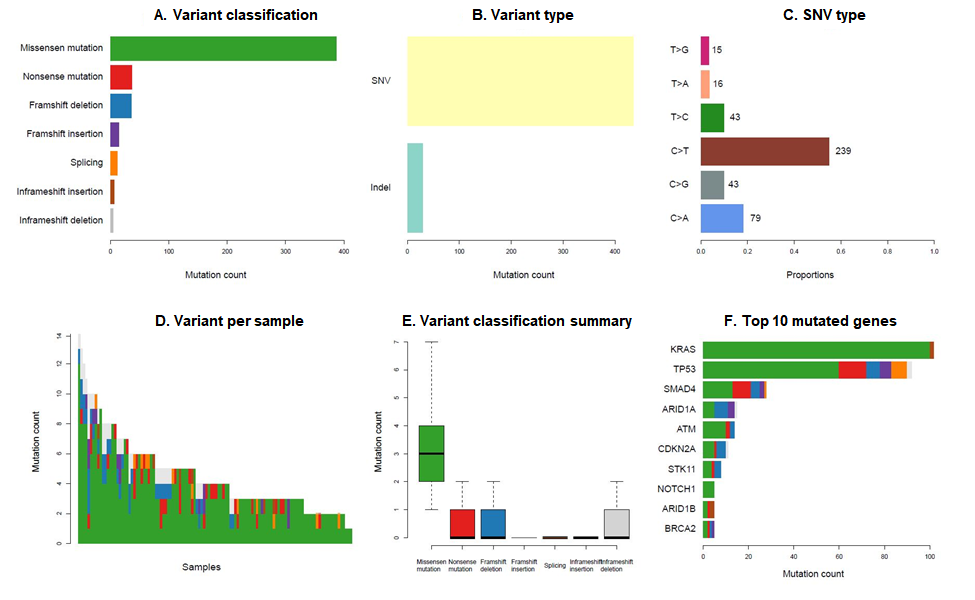


**Figure S2. Additional genomic alterations identified by targeted deep sequencing in EUS-FNB specimens of patients with PDAC.** (A) variant classification, (B) Variant types, (C) Single nucleotide variant (SNV) types, (D) Variants per sample, (E) Variant classification summary, (F) Top 10 mutated genes.


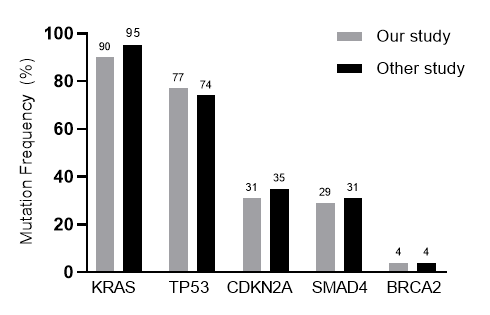


**Figure S3.** Comparison of mutation frequency of main genetic drivers in PDAC.For main genetic drivers in PDAC carcinogenesis, mutation frequency in our study were compared with that in other study [[7](#_ENREF_7)].
