# Supplementary material for: Accurate Prognosis Prediction of Pancreatic Ductal Adenocarcinoma Using Integrated Clinico-Genomic Data of Endoscopic Ultrasound-Guided Fine Needle Biopsy
Source: Cancers (Basel). 2021 Jun 3;13(11):2791. doi: 10.3390/cancers13112791 (PMC8199936; doi:10.3390/cancers13112791)
Supplement: Supplementary file 1 [file cancers-13-02791-s001.zip › cancers-1199860-supplementary/supplementary figures.pdf]

Supplementary Materials:

Accurate Prognosis Prediction of Pancreatic Ductal Adenocarcinoma using Integrated Clinico-Genomic Data of Endoscopic Ultrasound-Guided Fine Needle Biopsy

Table S1. DNA QC.

Table S2. Sequencing QC stat.

Table S3. CancerSCAN gene list.

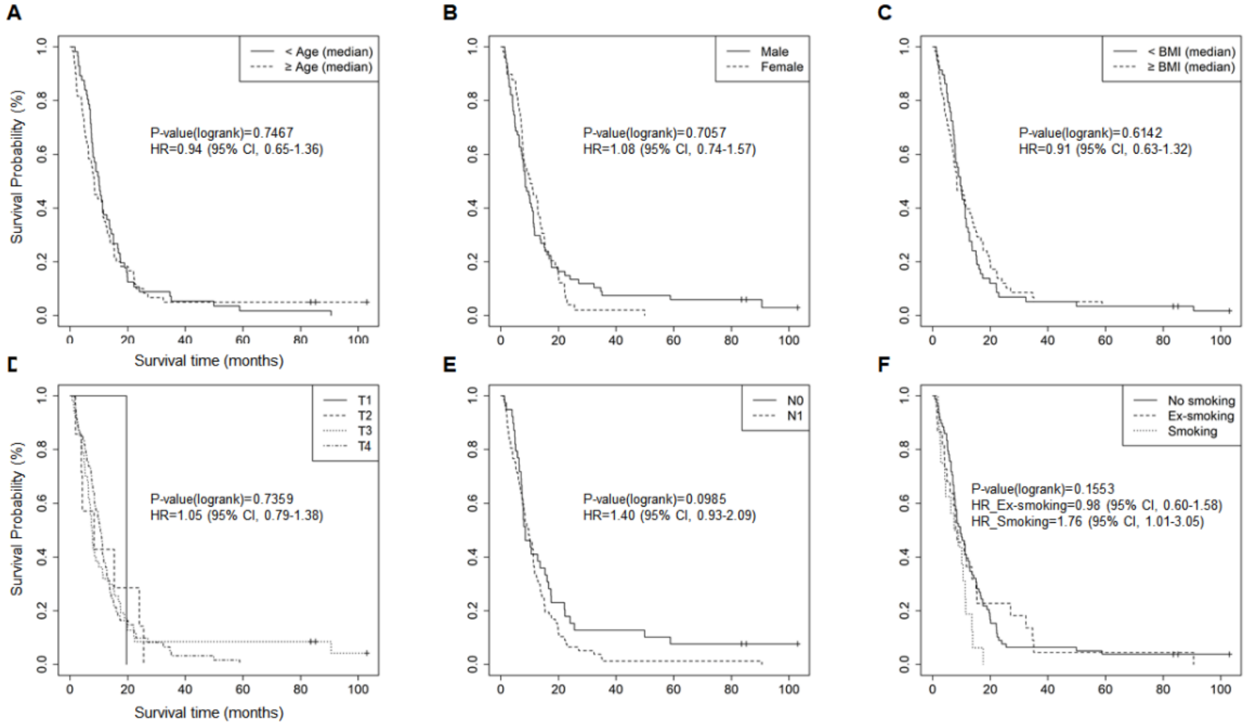

Figure S1. Kaplan-Meier plots for clinical factors not significantly associated with survival. The Kaplan-Meier plots demonstrate clinical factors without a significant ($P<0.05$) association with the survival of patients with PDAC. (A) Age, (B) Sex, (C) BMI, (D) T classification (size and direct extent of the primary tumor), (E) N classification and (F) Chemotherapy. Hazard ratio (HR), confidence interval (CI), and P value were obtained from Univariate Cox proportional hazard test.

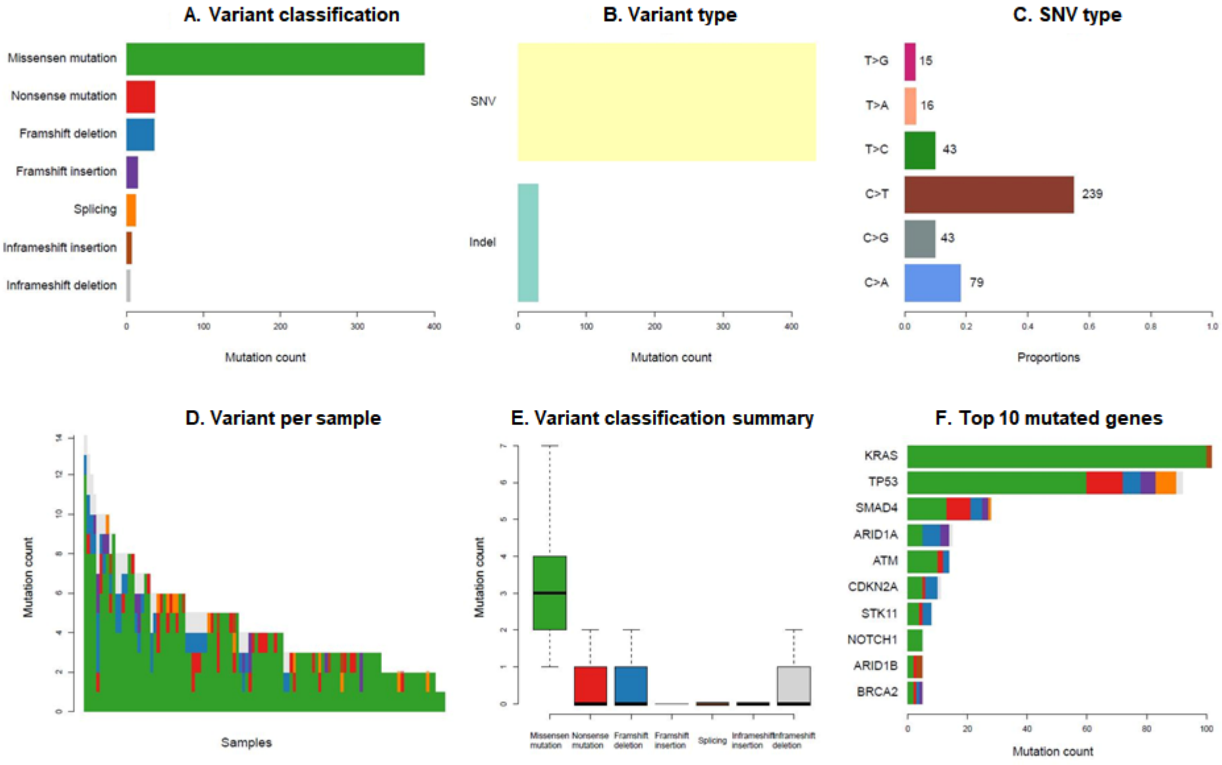

Figure S2. Additional genomic alterations identified by targeted deep sequencing in EUS-FNB specimens of patients with PDAC. (A) variant classification, (B) Variant types, (C) Single nucleotide variant (SNV) types, (D) Variants per sample, (E) Variant classification summary, (F) Top 10 mutated genes.

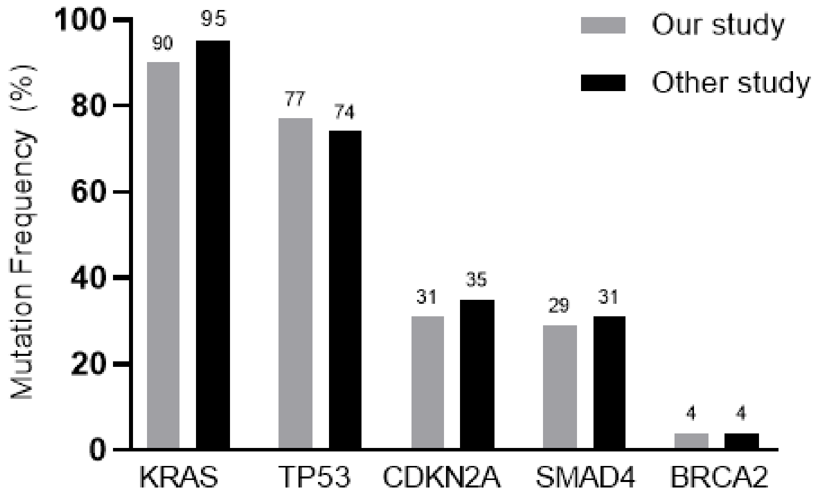

Figure S3. Comparison of mutation frequency of main genetic drivers in PDAC.For main genetic drivers in PDAC carcinogenesis, mutation frequency in our study were compared with that in other study [7].
